# Pollen sensitisation profiles of allergic patients in a Central European region

**DOI:** 10.1186/2045-7022-4-S2-P38

**Published:** 2014-03-17

**Authors:** Petr Panzner, Tomas Vlas, Martina Vachova, Petra Vitovcova

**Affiliations:** 1 Dept. of Immunology and Allergology, Medical Faculty in Pilsen, Charles University Prague, Alej Svobody 80, Pilsen, 30460, Czech Republic; 2 Charles University Prague, Medical Faculty in Pilsen, Dept. of Immunology and Allergology, Pilsen, Czech Republic

## Objective

The aim of our study was to assess the pollen sensitization patterns by means of molecular diagnosis approach in the region of Pilsen, Czech Republic.

## Methods

The microarray system ImmunoCAP ISAC has been used for specific IgE detection to 113 different allergenic molecules. Sera from 789 patients sensitized to at least one pollen-derived molecule were subject of analysis. These patients suffered from at least one of the following diagnoses: chronic rhinitis (62%), bronchial asthma (33%), atopic dermatitis (28%), urticaria or angioedema (28%) and/or anaphylaxis (11%). Patient age ranged from 2 to 68 years, with a mean age of 32,4 years. The sex ratio was 37% men to 63% women.

## Results

The most frequent sensitization rate was observed to grass-derived species-specific molecules (82,2% overall), the most frequent being Phl p 1 (66,3%), markedly overwhelming sensitization rates to any non-pollen-derived molecule. The second one were pollen-derived PR-10 molecules (53,2% overall), of which the large majority included Bet v 1 (52,3%). Sensitization to these two types of pollen components (and their co-sensitizations with other components) forms the vast majority of pollen sensitizations. The patterns of co-sensitization is presented by means of Venn diagram approach. Sensitization to Cupressaceae-derived molecules was observed in 16,0% of subjects, to Oleaceae derived-molecules in 12,5% (Ole e 1 and Ole e 9 in 9,0% and 3,8% resp.) and to the plane tree-derived molecules Pla a 2 and Pla a 3 in 15,4% and 4,1% resp; these relatively high rates were surprising as the respective pollens have not been considered as important in the region. The sensitization rates for further molecules were: Art v 1 - 13,4%, Pla l 1 - 11,4%, Che a 1 - 9,9%, Par j 2 - 0,9%, Sal k 1 - 0,6% and Amb a 1 - 0,3%. The sensitization rates to cross-reacting molecules were generally not as high as reported from other regions (profilins 16,2%, polcalcins 5,5%, LTPs 6,7%). Only 1,8% patients reacted to pollen-derived panallergen and not simultaneously to a pollen species-specific component.

## Conclusion

Molecular diagnosis of allergy gives a more precise and comprehensive insight into pollen sensitization patterns than extract-based testing, allowing for better understanding of the sensitization process and regional differences. The data presented may help to improve diagnostic and treatment specific procedures in the respective region.

